# Different Mechanisms of Inflammation Induced in Virus and Autoimmune-Mediated Models of Multiple Sclerosis in C57BL6 Mice

**DOI:** 10.1155/2013/589048

**Published:** 2013-08-28

**Authors:** Abhinoy Kishore, Anurag Kanaujia, Soma Nag, A. M. Rostami, Lawrence C. Kenyon, Kenneth S. Shindler, Jayasri Das Sarma

**Affiliations:** ^1^Department of Biological Sciences, Indian Institute of Science Education and Research-Kolkata, Mohanpur, Nadia West Bengal 741252, India; ^2^IISER Mohali, Knowledge City, Sector 81, SAS Nagar, Mohali 140306, India; ^3^Department of Neurology, Thomas Jefferson University, Philadelphia, PA 19107, USA; ^4^Department of Pathology, Anatomy and Cell Biology, Thomas Jefferson University, Philadelphia PA 19107, USA; ^5^University of Pennsylvania, Scheie Eye Institute and F.M. Kirby Center for Molecular Ophthalmology, Philadelphia, PA 19104, USA

## Abstract

Multiple sclerosis (MS) is an inflammatory demyelinating disease of the human central nervous system (CNS). Neurotropic demyelinating strain of MHV (MHV-A59 or its isogenic recombinant strain RSA59) induces MS-like disease in mice mediated by microglia, along with a small population of T cells. The mechanism of demyelination is at least in part due to microglia-mediated myelin stripping, with some direct axonal injury. Immunization with myelin oligodendrocyte glycoprotein (MOG) induces experimental autoimmune encephalomyelitis (EAE), a mainly CD4^+^ T-cell-mediated disease, although CD8^+^ T cells may play a significant role in demyelination. It is possible that both autoimmune and nonimmune mechanisms such as direct viral toxicity may induce MS. Our study directly compares CNS pathology in autoimmune and viral-induced MS models. Mice with viral-induced and EAE demyelinating diseases demonstrated similar patterns and distributions of demyelination that accumulated over the course of the disease. However, significant differences in acute inflammation were noted. Inflammation was restricted mainly to white matter at all times in EAE, whereas inflammation initially largely involved gray matter in acute MHV-induced disease and then is subsequently localized only in white matter in the chronic disease phase. The presence of dual mechanisms of demyelination may be responsible for the failure of immunosuppression to promote long-term remission in many MS patients.

## 1. Introduction

 Multiple sclerosis (MS), one of the most common neurological diseases of the central nervous system (CNS), is characterized by multifocal inflammation, demyelination, and axonal damage [1–3]. It is believed to be an autoimmune disease in which exposure of genetically predisposed people to environmental factors triggers a breakdown in T-cell tolerance to myelin antigens. Most studies have focused on the pathogenic role of myelin-specific CD4^+^ T cells because of the relatively strong association of susceptibility to MS with major histocompatibility complex (MHC) class II alleles [4–7], but there is also increasing recognition of the importance of CD8^+^ T cells in the pathogenesis of demyelination [8–10]. Conditions that lead to a loss of tolerance in myelin-specific T cells are not known. Viral infection has long been postulated to be an environmental trigger that contributes to the etiology of MS [11–14], although no specific virus has been confirmed as being a causative agent. 

 To better understand the structural-morphological diversity of MS, many studies use experimental autoimmune encephalomyelitis (EAE) induced by the MBP-PLP fusion protein MP4, MOG peptide 35–55, or PLP peptide 178–191 in mice, which, respectively, display distinct pathologies [15]. Major differences between the models reside in regional/tract specificity, kinetics of demyelination, and motor neuron involvement. All three models of induced EAE in mice provide a reasonable strategy for reproducing distinct adaptive immune-mediated pathologic features of demyelination. Previous studies [15] showed that the MP4 model is characterized by coinfiltration of B cells, CD8^+^, CD4^+^ T cells, dendritic cells, and macrophages, whereas in MOG- and PLP-induced models, CD4^+^ T cells and macrophages were the predominant cell types. It is generally accepted that in different EAE models, CD4^+^ T cells and macrophages are the predominant lesional cell types, while CD4^+^ T cells are the main effector cells. 

 Similarly, a few animal models exist in which viral infection triggers CNS demyelination such as Theiler's murine encephalomyelitis virus (TMEV) [16, 17] and neurotropic strains of mouse hepatitis virus (MHV) [18–21]. Chronic viral-induced demyelination is associated with viral persistence [22, 23] and concomitant upregulation of major histocompatibility complex class I antigens [24–28]. While studies suggest that an intact adaptive immune system is required to promote demyelination in many viral induced models, one neurotropic strain of MHV, MHV-A59, promotes demyelination even in the absence of B and T cells [29]. Furthermore, depletion of CD4^+^ or CD8^+^ T cells after the acute phase of infection does not prevent demyelination [30]. Thus, different related strains of MHV may induce demyelination via unique mechanisms, and it is likely that in the absence of an intact immune response, CNS infection with some strains of MHV is responsible for onset of axonal loss and demyelination, possibly through direct destruction of CNS cells [31]. 

 While EAE is useful in dissecting the role of T-cell- mediated myelin damage, viral models are helpful in understanding direct CNS cellular injury and demyelination that does not require an intact immune system. EAE- and MHV-induced demyelinating diseases are widely used MS models. Such studies have utilized that differing genetic backgrounds, or similar genetic backgrounds may have been used but in different laboratories or at different times, thus limiting direct comparison of the models. While some similarities and differences in mechanisms of demyelination have been noted, it is not clear whether differences are due to the disease itself or due to genetic or environmental differences. In the current study, we compared EAE- and RSA59- (isogenic recombinant strain of MHV-A59)-induced neuroinflammation in the identical genetic background (C57BL/6) at the same time and in the same laboratory and directly compared the type and pattern of CNS inflammation.

## 2. Materials and Methods

### 2.1. Mice

Four-week- and eight-week-old virus-free C57BL/6 mice were purchased from the Jackson Laboratory (Bar Harbor, ME, USA). All animal procedures and care were conducted in accordance with ethical guidelines approved by the Institutional Animal Care and Use Committee.

### 2.2. Induction of EAE and Scoring of Clinical Symptoms

Eight-week-old C57BL/6 mice were injected subcutaneously with 100 *μ*g MOG35–55 peptide (MEVGWYRSPFSRVVHLYRNGK) [32, 33] in complete Freund's adjuvant containing 4 mg/mL *Mycobacterium tuberculosis *H37Ra (DIFCO, Michigan, USA) at two sites on the back. 200 ng pertussis toxins were given intraperitoneally on days 0 and 2 after immunization. Mice were scored daily for neurologic dysfunction according to a 0–5 scale as follows: partial limp tail, 0.5; full limp tail, 1; limp tail and waddling gait, 1.5; paralysis of one hind limb, 2; paralysis of one hind limb and partial paralysis of the other hind limb, 2.5; paralysis of both hind limbs, 3; ascending paralysis, 3.5; weakness of the upper limb, 4; moribund, 4.5; death, 5 [32, 33]. At different days after immunization, mice were sacrificed, and tissues were harvested for histology.

### 2.3. Viruses

RSA59, an isogenic recombinant strain of MHV-A59, was used as previously described [31, 34, 35]. RSA59 expresses enhanced green fluorescence protein (EGFP) which facilitates detection of viral antigen by fluorescence without tissue staining [36]. Virulence was assessed in previous studies by calculating the lethal dose that killed 50% of mice (LD_50_). Mice were injected intracranially (i.c.) with serial 10-fold dilutions of viruses (five mice per dilution). Signs of disease or death were monitored on a daily basis up to 30 days after infection. LD_50_ values were calculated by the Reed-Muench method [37]. 

### 2.4. Inoculation of Mice

Four-week-old, MHV-free, C57BL/6 (B6) mice (Jackson Laboratory) were inoculated intracranially with 50% LD_50_ dose of RSA59 strain (20,000 PFU) as described previously [36]. 

Mice were monitored daily for signs of disease. Mock-infected controls were inoculated similarly but with an uninfected cell lysate at a comparable dilution. Animals were sacrificed (five mice per group) at day 3, 5, 7, and 30 after infections.

### 2.5. Estimation of Viral Replication

The efficiency of replication of the RSA59 was determined in mice inoculated intracranially at the designated dose. On days 1, 3, 5, and 7 after infection, mice were sacrificed and perfused with 20 mL of PBS, and the brains and livers were removed. The left halves of the brain were placed directly into 2 mL of isotonic saline with 0.167% gelatin (gel saline). The remainder of the brain and liver was fixed in 4% paraformaldehyde (PFA) and processed for histology. All organs collected for viral titer were weighed and stored frozen at −80°C until titered for virus. Brain and liver tissues from infected and control mice were homogenized, and viral titers were determined by plaque assay on an L2 cell monolayer [38–40]. 

### 2.6. Histopathology

MOG-immunized or viral-infected mice were sacrificed and perfused transcardially with 40 mL of PBS followed by PBS containing 4% PFA. Brain and spinal cord tissues were collected, postfixed in 4% PFA overnight at room temperature (RT), and embedded in paraffin. 5 *μ*m sections were processed and stained with Hematoxylin and Eosin (H&E) for assessment of inflammation and Luxol fast blue (LFB) for demyelination. Spinal cord histopathology was assessed as in prior studies [32, 35]. Briefly, two sections were examined from each of three spinal cord levels (cervical, thoracic, and lumbar) for each mouse. All slides were coded and read in a blinded manner. Sections were assessed as follows; inflammation: 0, none; 1, few inflammatory cells; 2, organization of perivascular infiltrates; and 3, increasing severity of perivascular cuffing with extension into the adjacent tissue. Demyelination was scored based on detection of focal white matter areas lacking LFB staining using the relative four-point scale; 0, none; 1, rare foci; 2, a few areas of demyelination; 3, large (confluent) areas of demyelination; 3.5, large confluent areas in different quadrants of spinal cord white matter; 4, loss of myelin from all quadrants of the spinal cord. To confirm expected virulence of the strains used, livers from the infected mice were embedded in paraffin, sectioned at 5 *μ*m, and stained with H&E [39, 41].

### 2.7. Immunohistochemical Analysis

Serial sections from spinal cords were stained by the avidin-biotin-immunoperoxidase technique (Vector Laboratories) using 3, 3-diaminobenzidine as substrate, and a 1 : 100 dilution of anti-CD45 (LCA; leukocyte common antigen, LY-5, BD Pharmingen), anti-Iba1 (Wako, Richmond, VA, USA), or CD3 (Dako; Carpinteria, CA, USA) as primary antibodies. Control slides from mock-infected mice were incubated in parallel.

## 3. Results and Discussion

### 3.1. Classical Clinical Symptoms in EAE Mice

**Figure 1:**
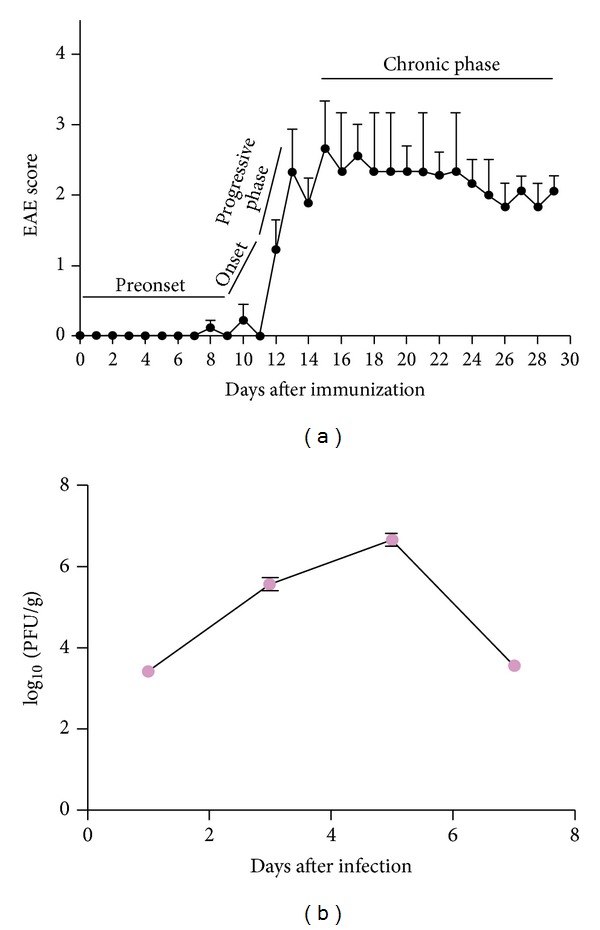
(a) Clinical profile of EAE. Female C57BL/6 mice (*n* = 9) were immunized with MOG 35–55 and scored daily using a previously reported 5-point scale of ascending paralysis [32, 33]. Data represent mean and standard error of the mean (SEM) generated from multiple animals in one experiment. One representative experiment of three is shown. (b) RSA59 replication in the brains of mice. C57BL/6 mice were infected with 20,000 PFU of RSA59 by intracranial inoculation. Mice were sacrificed at days 1, 3, 5, and 7 after inoculation, and viral titers were determined by plaque assay as in prior studies [36, 39]. The data represent the means (and standard deviations) of the titers from five mice. Titers are expressed as log_10_ PFU per gram of tissue.

Evidence suggests that immunization of mice with MOG to induce EAE results in an encephalitogenic T-cell response and a demyelinating autoantibody response. Immunization of MOG35-55 peptide together with *Mycobacterium tuberculosis* and pertussis toxin results in a chronic progressive form of EAE in C57BL/6 mice. In the current studies, 8-week-old C57BL/6 mice (*N* = 30) were immunized with MOG35–55 and were monitored daily for signs and symptoms of EAE for 30 days. Mice developed the classical chronic progressive clinical profile of EAE (Figure 1(a)). Onset of disease occurs between days 9–12. Mice were sacrificed at EAE onset, the progressive phase (days 13–15) and at the peak of disease (days 16–30). Remaining mice (*n* = 9) were kept for scoring of neurologic symptoms throughout the 30 days. 

### 3.2. Viral Virulence and Replication in the Brain

Four-week-old, MHV-free, C57BL/6 (B6) mice were inoculated intracranially with 50% LD_50_ dose of isogenic recombinant strain RSA59 strain (20,000 PFU) as described previously. Mice were monitored daily for signs of disease. Mock-infected controls were inoculated similarly but with an uninfected cell lysate at a comparable dilution. The efficiency of replication of RSA59 was determined in mice inoculated intracranially, and the peak of viral replication was at day 5, similar to prior studies (Figure 1(b)).

### 3.3. Comparative Analysis of Disease Symptoms

Mice immunized with MOG displayed classic features of EAE including tail limpness, waddling gait, hind limb paralysis and ascending paralysis. In contrast, MHV-infected mice do not manifest obvious clinical neurologic impairment. Instead, they typically demonstrate ruffled fur, hunched back posturing, and significant weight loss (data not shown). 

### 3.4. Typical CNS Inflammation in EAE Mice

**Figure 2:**
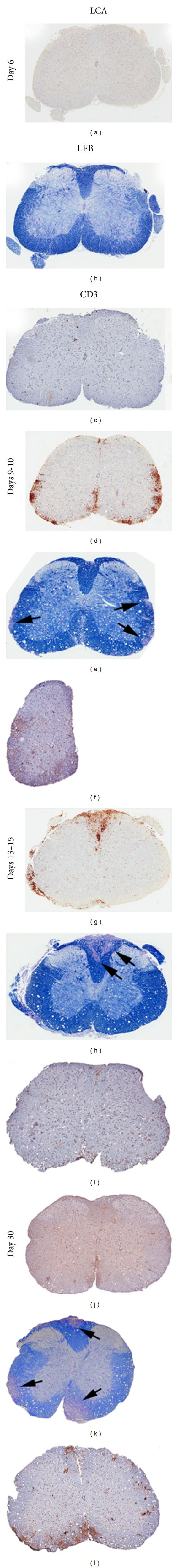
Comparative histopathology of EAE spinal cords at different days after immunization. Representative cross-sections of spinal cords (5 *μ*m thick) of EAE mice at different days after immunization were stained with Luxol fast blue (for myelin) and immunohistochemically for CD45 (LCA for inflammatory cells) and CD3 (for T cells). A hemi cord is shown in (f). ((a)–(c)) Day 6 after immunization (before EAE onset). ((d)–(f)) Days 9-10 after immunization (EAE onset). ((g)–(i)) Days 13–15 after immunization (progressive phase), ((j)–(l)) Days 30 after immunization (chronic phase). ((a), (d), (g), (j)) LCA stain. ((b), (e), (h), (k) Luxol fast blue. ((c), (f), (i), (l)) CD3. In comparison to day 6 spinal cords, days 9-10 cords demonstrate an influx of LCA and CD3 positive cells in the white matter. Luxol fast blue stain identifies early demyelinating plaques in the white matter. During the progressive phase, there is persistent LCA immunoreactivity, whereas CD3 is decreased. The size of demyelinating plaques increases. At later times (chronic phase), LCA immunoreactivity decreases, while CD3 increases. Demyelinating plaques increase in number and size. Arrows mark locations of demyelinating plaques.

**Figure 3:**
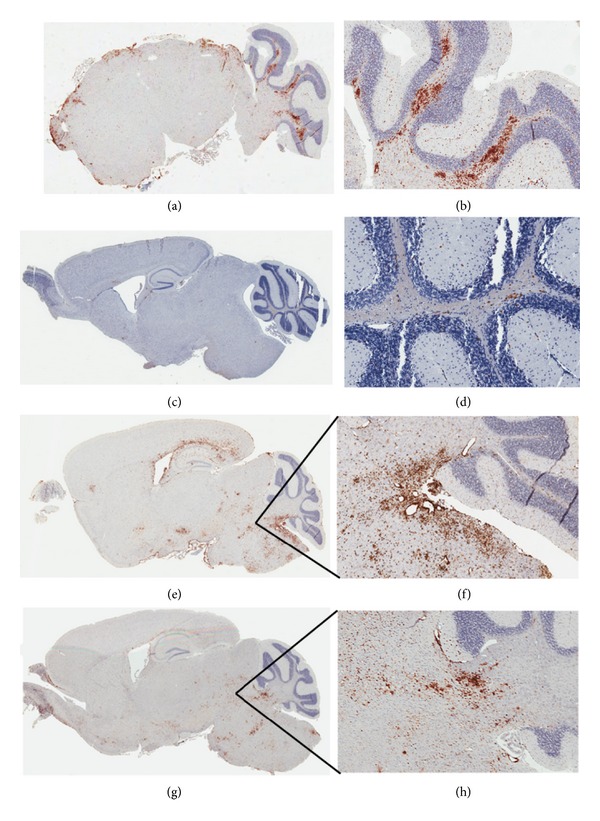
Brain inflammation in EAE and RSA59-infected mice. EAE ((a)–(d)): mid-sagittal sections of brains (5 *μ*m thick) at early and late phases after immunization were stained immunohistochemically for CD45 (LCA). ((a), (b)) day 9 after immunization. ((c), (d)): day 30 post immunization. There is mostly leptomeningeal (surface) and deep cerebellar white matter LCA immunoreactivity at early phases that decrease dramatically but remains in the same locations at late time points. ((a), (c)) Scanned images. ((b), (d)) Higher magnification of corresponding deep cerebellar white matter (100x). RSA59-infection ((e)–(h)) mid-sagittal sections of brains (5 *μ*m thick) at early and late phases after inoculation were stained immunohistochemically with CD45 (LCA). ((e), (f)) Day 7. ((g), (h)) Day 30. At early phases post-inoculation, there are both white and gray matters' infiltration by LCA immunoreactive cells that diminishes in intensity over time and is mainly observed in deep cerebellar white matter at day 30. ((e), (g)) Scanned images. ((f), (h)) Higher magnification of corresponding deep cerebellar white matter (100x). Black lines in (e) and (g) show areas magnified in (f) and (h).

Histological analysis of EAE spinal cords was done as in prior studies [32, 33] and showed expected mononuclear cell infiltration (leukocyte common antigen (LCA) staining), demyelination (Luxol fast blue (LFB) staining), and T-cell infiltration (CD3 staining) at all time points examined after disease onset (Figure 2). Similar inflammation was present in other parts of the CNS, including the meninges of the brain and deep cerebellar white matter (Figure 3). CNS inflammation was present throughout the disease course, and levels of demyelination progressed over time (Figure 2; Table 1). At all time points, infiltrating inflammatory cells (LCA^+^ and CD3^+^) in EAE spinal cords were restricted to white matter, without significant gray matter inflammation (Figure 2; Table 2). Overall, LCA staining increased by days 10–13 and then decreased by day 30. CD3^+^ cells initially increased then decreased by days 12–15 only to rebound and localize to demyelinating plaques in the chronic phase. Average demyelination scores are shown in Table 1.

### 3.5. Inflammatory CNS Pathology of RSA59

**Figure 4:**
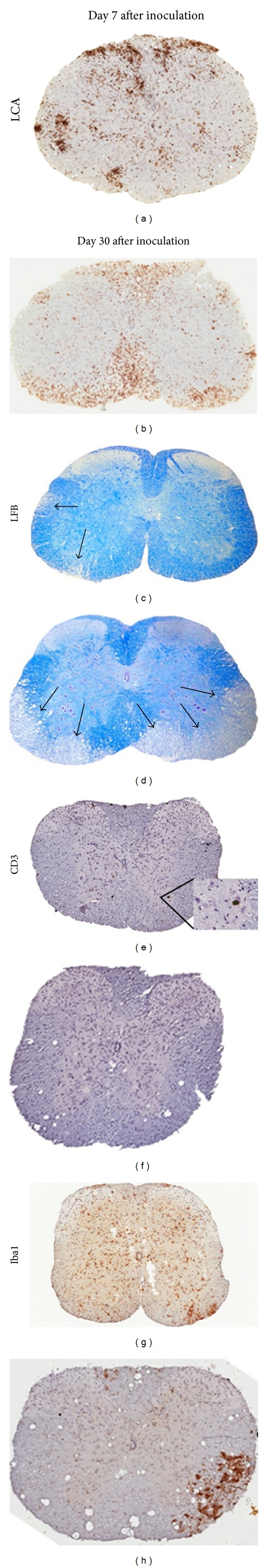
Spinal cord inflammation and demyelination in RSA59 infection. Representative cross-sections of spinal cords (5 *μ*m thick) of virally infected mice at different days after inoculation were stained with LFB (for myelin) and immunohistochemically for CD45 (LCA), CD3 (T cells), and Iba1 (macrophages/microglia). ((a), (c), (e), (g)) Day 7 after inoculation (peak of inflammation). ((b), (d), (f), (h)) Day 30 after inoculation (late phase). ((a), (b)) LCA stain. ((c), (d)) Luxol fast blue. ((e), (f)) CD3. ((g), (h)) Iba1. At day 7 after inoculation, both gray and white matter are infiltrated by LCA positive cells, whereas by day 30, LCA immunoreactivity is predominately localized to the white matter. In contrast, there are few CD3 positive cells at both early and late phases. Iba1 immunoreactivity increases and becomes progressively localized to demyelinating plaques. Inset in (e) shows higher magnification of rare CD3 immunoreactive lymphocytes. LFB stains demonstrate increasing numbers and sizes of demyelinating plaques over time. Arrows mark locations of demyelinating plaques.

Pathology was assessed in sagittal brain sections and cross-sections from each spinal cord level at day 7 (peak inflammation) and day 30 (peak demyelination) after infection with RSA59. Demyelinating plaques were detected by LFB staining at day 7, and spinal cords showed a similar pattern of demyelination at day 30, but the number and area of plaques was larger (Figures 4(c) and 4(d)). Average demyelination is shown in Table 1. In contrast to EAE, LCA^+^ cells are present in both gray and white matters of brain and spinal cord during acute MHV infection, but by day 30, they localize to white matter tracts, especially in spinal cord and deep cerebellar white matter (Figures 3 and 4; Table 2). In spinal cord, at days 3 and 5, most LCA^+^ are Iba-1^+^(microglia/macrophage marker), and these cells are present in gray matter (data not shown), and by day 7, LCA^+^/Iba-1^+^ cells localize in both gray and white matters (Figure 4; Table 2). By day 30, LCA^+^ and Iba1^+^ cells are present mainly in demyelinating plaques. In addition to the change in distribution of LCA^+^ cells, there is also a change in staining intensity and morphology. At early phases (Figure 4(a)), cells are compact and darkly staining, whereas at late phases (Figure 4(b)), cells are more ramified and less intensely stained, reflecting a change from newly arrived monocytes to mature activated macrophages. Data demonstrates that in viral-infected spinal cord there is an increase of Iba1^+^ cells; however, the distribution of these inflammatory cells varies significantly at different days after infection. Interestingly, in contrast to EAE, CD3^+^ cells are virtually absent.

## 4. Conclusion

 Demyelination was a prominent and consistent finding, with 100% of MHV-infected mice and 85% of EAE mice developing demyelination. Though the gross level of inflammation was similar between the two models, significant differences were observed in the pattern of CNS inflammation. In EAE, inflammation was observed only in white matter at all phases of disease (Table 2). This is not unexpected since the myelin antigenic target is concentrated in white matter. In contrast, almost all MHV-infected mice exhibited inflammation within gray matter during the acute phase of disease, with inflammation becoming restricted to white matter in the chronic phase (Table 2). This pattern is consistent with the neuronal tropism of demyelinating strain of MHV and its subsequent axonal transport into white matter reported previously [31, 40]. Findings are consistent with prior studies suggesting that CNS tissue injury in MHV infection is mediated both by viral-induced cytotoxicity and by immune mechanisms. The relative contributions of these two components differ depending on viral tropism, rate of viral spread, and specificity of the immune response [40]. Recent neural cell tropism studies indicate that axonal transport of MHV from gray to white matter is necessary to induce demyelination [31, 34]. Results show how direct autoimmune inflammation along CNS white matter tracts and inflammation secondary to viral infection tracking from gray matter to white matter can both produce similar chronic white matter demyelinating plaques. Comparison of EAE with RSA59-induced demyelination provides insight into mechanisms operative in such a complex disease as MS.

## Figures and Tables

**Table 1:** Average demyelination score in RSA59-infected mice and EAE mice.

	No. of mice	No. of sections	% mice with demyelination	Mean score of demyelination (mean ± SD)	*P* values
Acute phase RSA59 (day 7)	10	60	100%	0.60 ± 0.46	*P* < 0.0010
Chronic phase RSA59 (day 30)	12	72	100%	2.0 ± 0.40	*P* < 0.0001
Acute phase EAE (days 8–15)	20	100	85	1.88 ± 0.99	*P* < 0.0005
Chronic phase EAE (day 30)	10	60	80	2.25 ± 0.85	*P* < 0.0001

**Table 2:** Pattern and level of inflammation in EAE- and RSA59-infected mouse spinal cords.

Distribution of inflammation	Acute phase MHV	Chronic phase MHV	Acute phase EAE	Chronic phase EAE
Gray matter	1/8 (12.5%)	0/5 (0%)	0/8 (0%)	0/12 (0%)
White matter	1/8 (12.5%)	5/5 (100%)	8/8 (100%)	12/12 (100%)
Both gray and white matters	6/8 (75%)	0/5 (0%)	0/8 (0%)	0/12 (0%)
No inflammation	0/8 (0%)	0/5 (0%)	0/8 (0%)	0/12 (0%)

*X*/*Y* = number of mice exhibiting the staining pattern (*X*) out of the total number examined (*Y*). Numbers in parentheses are the percentages of mice exhibiting each staining pattern.
